# Automated detection of repetitive focal activations in persistent atrial fibrillation: Validation of a novel detection algorithm and application through panoramic and sequential mapping

**DOI:** 10.1111/jce.13752

**Published:** 2018-10-14

**Authors:** Shohreh Honarbakhsh, Richard J. Schilling, Rui Providencia, Emily Keating, Simon Sporton, Martin Lowe, Pier D. Lambiase, Anthony Chow, Mark J. Earley, Ross J. Hunter

**Affiliations:** ^1^ Barts Heart Centre, Barts Health NHS Trust London UK

**Keywords:** atrial fibrillation, atrial tachycardia, CARTOFINDER mapping, catheter ablation, focal drivers, rotors

## Abstract

**Introduction:**

Identifying drivers in persistent atrial fibrillation (AF) remains challenging. We sought to validate an automated system for detection of focal activation using basket and PentaRay catheters in AF.

**Methods:**

Patients having ablation for atrial tachycardia (AT) and persistent AF were mapped. Thirty‐second unipolar basket and PentaRay recordings were analyzed using CARTOFINDER. Focal activation or “region of interest” (ROI) was defined as more than or equal to 2 consecutive focal activations with one electrode leading relative to its neighbors with QS morphology on the unipolar electrogram. ROI was validated in AT. AF patients were mapped to (1) look for evidence of focal activations on wavefront maps, (2) evaluate whether these were detected as ROI on basket recordings, and (3) whether these sites could be identified on sequential PentaRay recordings.

**Results:**

ROIs were identified in five focal ATs but none of 16 reentrant ATs. Twenty‐eight AF patients had 35 focal drivers identified from basket wavefront maps with an ablation response in all (16 cycle length slowing and 19 AF termination). Thirty focal activations were detected on basket ROI maps (86%). Twenty‐three of 28 patients had sequential PentaRay mapping and 22 of 30 focal drivers in these patients (73%) were identified as ROI. These drivers had greater temporal stability (3.6 ± 0.6 vs 2.7 ± 0.6; *P* < 0.001), higher recurrence rate (12.4 ± 2.7 vs 7.2 ± 0.9; *P* < 0.001), and more frequently were associated with AF termination ( *P* < 0.001) compared with those not identified as ROI.

**Conclusions:**

Focal activations can be detected in AF using sequential recordings. The ablation response at focal sources suggests they may be viable therapeutic targets.

## 1 INTRODUCTION

The chaotic nature of persistent atrial fibrillation (AF) has hindered attempts to identify drivers through sequential mapping. Global mapping of the left atrium (LA) using different techniques has identified focal drivers and drivers with rotational activity.^1^, ^2^, ^3^, ^4^ Evaluation of global mapping data to identify intermittent drivers can be time consuming and subjective. Automated detection of drivers may facilitate and standardize interpretation of global mapping data. Furthermore, once validated, it may be possible to apply a system of automated detection to sequential mapping.

A novel algorithm has been developed as part of the CARTOFINDER mapping system that aims to detect repetitive focal activations automatically. Relevant areas are then highlighted as a “region of interest” (ROI). However, this algorithm has not been tested previously and to date no other system has utilized sequential mapping for automated detection of focal drivers in AF in vivo.

The aim of this study was to (1) validate the CARTOFINDER ROI algorithm in the automated detection of focal activations by mapping focal atrial tachycardias (AT), (2) apply this to persistent AF and correlate sites identified as ROI with focal drivers identified on global activation mapping with basket catheters, and (3) test the application of this system to sequential recordings acquired using a PentaRay catheter, by again comparing the ROI identified to the sites of drivers identified on global mapping.

## 2 MATERIALS AND METHODS

Patients undergoing catheter ablation for persistent AF (< 24 months and no previous AF ablation) and AT were prospectively included. Patients provided informed consent for their involvement in this study, and it was approved by the UK National Research Ethics Service (16/LO/1379).

### 2.1 Electrophysiology procedure

All cases were performed with CARTO (Biosense Webster Inc., Diamond Bar, CA). LA geometries and detailed bipolar voltage maps were created using a 2‐6‐2 mm spacing PentaRay NAV catheter (Biosense Webster, Inc) with a color fill threshold of 5 mm aiming for complete LA coverage. Low‐voltage zones (LVZs) were defined as sites with a bipolar voltage less than 0.5 mV.^5^

### 2.2 CARTOFINDER global and sequential mapping

The CARTOFINDER mapping system (Biosense Webster, Inc) was used to create global dynamic activation maps of the LA using basket catheters with a minimum of two 30‐second unipolar recordings pre‐ and post‐pulmonary vein isolation (PVI) per patient with the catheter repositioned between recordings.

CARTOFINDER annotates atrial signals using a system called “wavelet analysis”^6^ and from this local activation times (LATs) are obtained for each electrode. CARTOFINDER filters low quality signals secondary to noise utilizing an algorithm that looks for a set of different parameters such as number of atrial beats, signal to noise ratio, signal amplitude and frequency analysis, and decide if there is enough data to be used for the annotation algorithm. If not these signals are not annotated, no timing is assigned and the electrode is ignored. The LATs are then used to create the global dynamic activation maps.^3^ Unipolar recorded using Wilson’s central terminal and signals were filtered at 2 to 240 Hz, with a notch filter at 60 Hz. No further filtering or processing was applied. Filtering was kept consistent between all patients.

CARTOFINDER was also used to map the LA with sequential PentaRay catheter recordings aiming for even LA coverage with a minimum of 10 recordings per patient. Care was taken to ensure the best possible electrode contact and spacing between the PentaRay catheter splines.

The main aim of the study was to map the repetitive focal activations in the LA. The right atrium was not mapped in any of these patients.

### 2.3 Ablation strategy

All patients underwent wide area circumferential ablation to achieve PVI using a Thermocool SmartTouch Surround Flow catheter (Biosense Webster, Inc). The global dynamic activation maps were then prospectively reviewed by the two operators with the aim to identify and ablate repetitive focal activations that were defined as more than or equal to 2 consecutive focal discharges with radial spread.^3^, ^7^, ^8^ As the focus of this study was focal drivers, rotational drivers were not scrutinized further in this analysis.

Ablation at the repetitive focal activation site, as guided by the global activation maps was performed 20 minutes post‐PVI to minimize the impact of PVI on the ablation response seen at the focal site. Ablation was delivered with a contact force of 5 to 40 g and a power of 30 to 40 W. Ablation was initially performed at the center of the focal site with further consolidating lesions around the center. Ablation was continued until (1) a predefined ablation response was achieved (termination of AF or cycle length [CL] slowing of ≥30 ms); (2) no signal was present at the driver site; or (3) 5 minutes of ablation had been performed at each site. Beyond isolating PVs and targeting repetitive focal activations no additional ablation was performed in AF. If the AF organized into an AT this was mapped and ablated.

AF CL was measured preablation and postablation over 30 cycles from the bipole pair with the clearest electrogram on the PentaRay catheter that was positioned in the LA appendage. In a patient who may have multiple potential drivers it would seem important to note any marked response to ablation. So as to note only a marked response to ablation a significant change in AF CL was defined as more than or equal to 30 milliseconds as we have used in other recent studies.^3^, ^8^

### 2.4 ROI analysis

ROI maps were processed offline using an automated driver detection method to identify repetitive focal activations. The automated method required (1) demonstration of a QS morphology on the unipolar recording; (2) the atrial signal with QS morphology needs to be earlier than its neighboring electrodes; and (3) completing two consecutive repetitions. The repetitive focal activations were referred to as ROI sites and were highlighted on the LA geometry.

(1) Validation of the ROI system in the detection of focal activations in AT
  ROI maps using basket catheters were created in patients undergoing AT ablation. Conventional LAT maps, entrainment and ablation response then verified the AT mechanism. The identification of an ROI site was related to the presence of a focal versus reentrant mechanisms of AT. In those with focal AT, the location of these ROI sites were compared with the sites of focal activity on activation mapping.
(2) Identification of ROI in AF during global and sequential mapping.

The ROI sites identified on maps created with the basket catheter were compared offline to the global activation maps that were used to guide ablation of repetitive focal activations. Two observers that were blinded to the findings of the global maps performed the analysis to confirm whether sites identified as ROI met the necessary criteria. The electrode identified as an ROI site was then correlated to the site of the focal driver identified and marked on CARTO as a “location only” point by the two operators live during cases and the sites were said to correlate if the they measured less than 1 cm center to center measured using the distance measurement tool on CARTO. ROI sites identified on sequential PentaRay recordings were likewise compared with focal driver sites that had been identified and ablated using global activation maps generated from basket recordings.

The characteristics of the focal drivers that were identified as ROI were compared to those that were not. The driver characteristics included (1) consistency, the proportion of global maps that show the same repetitive focal activation; (2) temporal stability, average consecutive repetitions of a focal activation during a 30‐second recording; (3) recurrence rate, number of times a focal driver met the definition of a driver during a 30‐second recording.

### 2.5 Outcomes

Patients were followed up at 3, 6, 9, and 12 months with 48 hours of ambulatory Holter monitoring at 6 and 12 months and further monitoring dictated by symptoms. Success was defined as freedom from AF or atrial tachyarrhythmias lasting more than 30 seconds off antiarrhythmic drugs after a 3‐month blanking period as per current guidelines.^9^

### 2.6 Statistical analysis

Statistical analyses were performed using SPSS (IBM SPSS Statistics, Version 24; IBM Corp, New York, NY). Continuous variables are displayed as mean ± standard deviation (SD) or median (range). Categorical variables are presented as a number and percentage. The Student *t* test or the Mann‐Whitney *U* test was used for comparison of continuous variables. Sensitivity and specificity was determined for the ROI algorithm in terms of its ability to identify focal drivers. *P* < 0.05 was deemed as significant.

## 3 RESULTS

Fifty patients were included (34 patients undergoing ablation for persistent AF and 16 for AT) (Table 1).

**Table 1:** Patient demographics

Baseline characteristics	AT patients, *n* = 16	AF patients, *n* = 34
Age, y, mean ± SD	59.8 ± 13.0	59.2 ± 10.5
Male, *n* (%)	10 (63)	24 (71)
Diabetes mellitus, *n* (%)	0	1 (3)
Hypertension, *n* (%)	7 (44)	9 (26)
TIA/CVA, *n* (%)	0	1 (3)
Ischemic heart disease, *n* (%)	0	2 (6)
Cardiac surgery, *n* (%)	1 (6)	0
Left ventricular EF ≥ 55%, *n* (%)	14 (88)	30 (88)
LA area, cm^2^, mean ± SD	25.4 ± 3.9	26.0 ± 3.8
Bipolar voltage, mV, mean ± SD	0.31 ± 0.10	0.45 ± 0.16
AF duration, mo, mean ± SD	–	14.6 ± 4.6
Previous ablation, *n* (%)		
AF	14 (88)	0
Cavotricuspid isthmus dependent flutter	2 (13)	1 (3)
Current medical strategy		
Calcium channel blocker	0	2 (6)
Beta‐blockers including sotalol	16 (100)	20 (59)
Amiodarone	0	23 (68)
Flecainide	1 (6)	1 (3)
Current anticoagulation strategy		
Warfarin	13 (81)	5 (15)
Direct‐acting oral anticoagulants	3 (19)	29 (85)

Abbreviations: EF, ejection fraction; TIA/CVA, transient ischemic attack/cerebrovascular attack.

### 3.1 Validation of the ROI system in the detection of focal activations in AT

**Figure 1:**
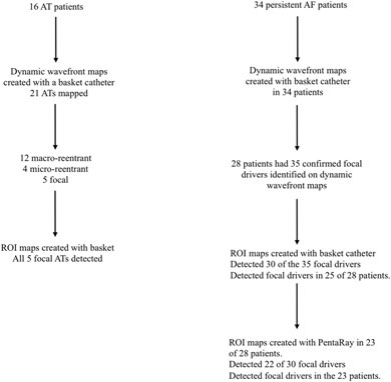
Flow diagram highlighting the mapping procedure in the persistent AF and AT patients and the number of drivers identified on the dynamic wavefront and ROI maps with simultaneous and sequential mapping. AF, atrial fibrillation; AT, atrial tachycardia; ROI, region of interest

**Figure 2:**
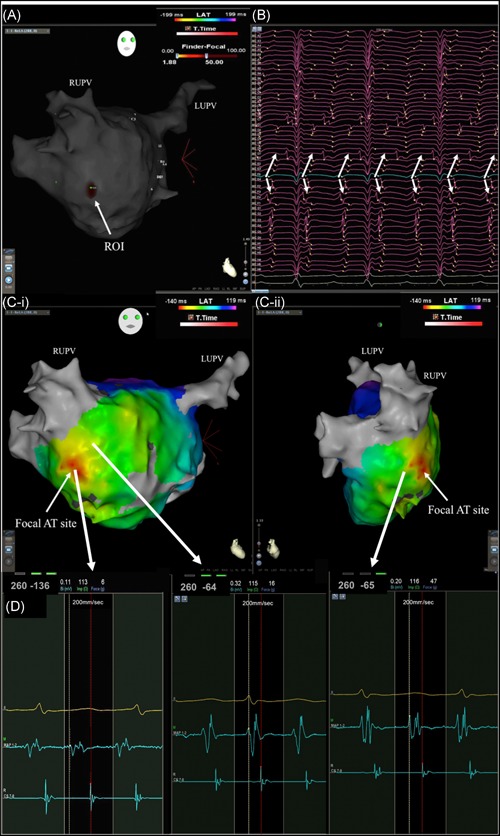
A focal AT mapped to the LA septum as indicated on (A) ROI map in a titled AP view created with simultaneous mapping with basket catheter. (B) Corresponding unipolar electrograms with the green electrode highlighting the ROI site and the arrows highlighting radial spread from this site. (Ci‐ii) conventional LAT map in a titled AP and right lateral view demonstrating the earliest activation site correlating to the ROI site. (D) Electrograms obtained from data points on CARTO during the creation of the LAT map with points on either side of the red area timing later. AP, anterioposterior; AT, atrial tachycardia; LA, left atrium; LAT, left atrial thrombus; LUPV, left upper pulmonary vein; ROI, region of interest; RUPV, right upper pulmonary vein

Twenty‐one ATs were mapped in the 16 AT patients (Figure 1) out of which five were focal (*n* = 2 anterior LA, *n* = 1 low posterior LA, *n* = 1 low anteroseptal LA, and *n* = 1 LA roof) in five patients. In these five patients, 15 simultaneous CARTOFINDER maps were created with a single ROI electrode identified on all maps correlating to the site of the focal AT as per the conventional LAT map (Figure 2A‐C). Manually reviewing the 7.5 minutes of unipolar recordings the criteria for ROI was consistently met as the AT cycle repeated itself. No ROI were identified on the 35 maps of the 16 microreentrant and macroreentrant ATs.

### 3.2 Mapping and ablation of repetitive focal activations in AF

In the 34 patients undergoing persistent AF ablation, 226 global activation wavefront maps were created in the LA (116 pre‐PVI and 110 post‐PVI, 3.3 ± 0.7 maps per patient at each stage).

Twenty‐eight of the 34 patients (82%) had a repetitive focal activation mapped and ablated as determined on 139 of the 226 (62%) global maps and 79 of the 110 post‐PVI global maps (72%). Thirty‐five repetitive focal activations were identified in the 28 patients (1.3 ± 0.4 per patient) with a predefined ablation response in all making them all drivers (20 repetitive focal activations [57%] resulted in AF termination in 20 patients [71%]) (Figure 1 and Supporting Information Table 2).

Focal drivers showed a mean of 11.1 ± 3.7 occurrences during a 30‐second recording and with 3.2 ± 0.8 consecutive repetitions at each occurrence. Drivers most often occurred for a mean of 2 to 3 consecutive repetitions (14/35, 40%). The proportion of drivers that occurred with a mean consecutive repetition of more than 3 to 4 and more than 4 to 5 were 16/35 (46%) and 5/35 (14%), respectively.

Drivers that showed greater recurrence rate (12.2 ± 3.1 vs 8.4 ± 2.1; *P* < 0.001) and higher temporal stability (3.6 ± 0.6 vs 2.7 ± 0.6; *P* < 0.001) were more commonly associated with AF termination compared with CL slowing. Focal drivers that were seen on pre‐PVI maps were also more commonly associated with AF termination compared to CL slowing (16 of 20 focal drivers resulting in AF termination were seen on pre‐PVI maps vs 5 of 15 drivers causing CL slowing were seen on pre‐PVI maps; *P* = 0.01). There was no significant difference in the LA appendage CL between cases that resulted in AF termination vs CL slowing with ablation (142.5 ± 22.3 vs 145.8 ± 32.4; *P* = 0.67).

Focal drivers were distributed throughout the LA but more commonly mapped to the roof (14 of 35, 40%) followed by the lateral wall (8 of 35, 23%). The drivers did not show a predilection to LVZs with equal distribution in LVZs (19 of 35, 54%) and non‐LVZs (16 of 35, 46%).

### 3.3 Identification of ROI in AF during global and sequential mapping

There was 100% interobserver agreement between the two operators regarding whether the ROI sites correlated with sites of focal activation on the global activation maps. Subsequent review by both operators showed that intraobserver agreement was also 100%.

#### 3.3.1 Global activation maps

**Figure 3:**
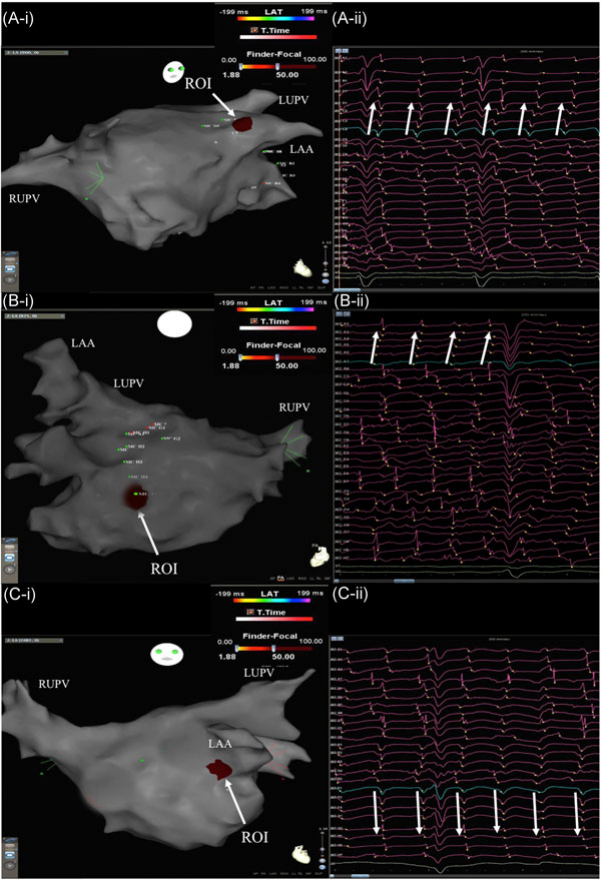
Demonstrates sites identified as ROI located at different LA anatomical surfaces in three different patients: (A) roof/LA appendage, (B) posterior‐inferior wall, (C) anterior LA appendage. Ablation at site (A) and (C) resulted in AF termination while at site (B) ablation resulted in CL slowing. It also shows the corresponding unipolar electrograms at the ROI site that confirms that all the sites meet the criteria of a ROI site (≥2 focal activations in which one electrode led relative to its neighbors with QS morphology on unipolar electrograms). LAA, left atrial appendage; LUPV, left upper pulmonary vein; RUPV, right upper pulmonary vein

**Figure 4:**
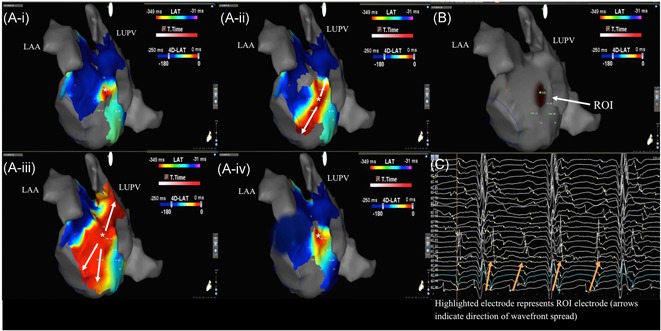
A focal driver mapped to the lateral wall on (Ai‐iv) still CARTOFINDER wavefront maps, (B) ROI map created with simultaneous mapping with basket catheter, and (C) corresponding unipolar electrograms recorded on the basket catheter with the green electrogram highlighting the ROI electrode. LAA, left atrial appendage; LUPV, left upper pulmonary vein; ROI, region of interest

ROI sites were identified on 121 of 139 global activation maps (87%) and on 58 of 79 post‐PVI global activation maps (79%). Twenty‐five of 28 patients (89%) had a ROI site identified. Manual analysis of the unipolar signals at the ROI site confirmed that these sites met the criteria of a ROI site (Figures 3A‐C and 4A‐C). ROI sites were not identified in the six patients in whom repetitive focal activations were not seen on the global maps. Supporting Information Table 1 shows details of each focal driver and whether it was detected as an ROI. Eight ROI maps (8/73, 11%) in four patients showed ROI sites that did not correlate to driver sites on global wavefront maps. These additional sites occurred in patients in whom the focal driver ablation response was CL slowing.

#### 3.3.2 Sequential activation maps

**Figure 5:**
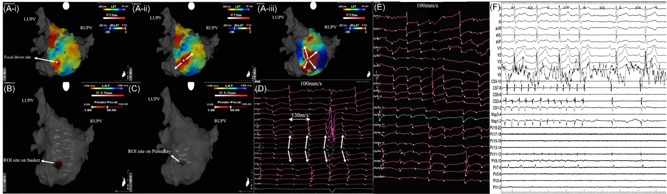
A focal driver mapped to inferior‐posterior wall on (Ai‐iii) still CARTOFINDER wavefront maps, (B) ROI map created with simultaneous mapping with basket catheter, (C) ROI map created with sequential mapping with PentaRay catheter, (D) corresponding unipolar electrograms recorded on PentaRay with the electrogram in green highlighting the electrode recording the initial signal at the focal driver site with radial spread to the surrounding electrodes. (E) Unipolar electrograms recorded on PentaRay between episodes of repetitive focal activations. (F) Electrograms demonstrating AF termination to sinus rhythm during ablation at the driver site. AF, atrial fibrillation; LUPV, left upper pulmonary vein; ROI, region of interest; RUPV, right upper pulmonary vein

Twenty‐eight of the 34 patients (82%) had sequential mapping performed with the PentaRay catheter with a total of 367 recordings (13.1 ± 1.6 recordings per patient). Out of the 28 patients that had a focal driver identified, 23 had sequential mapping performed (82%) corresponding to 303 recordings (13.2 ± 1.6 maps per patient). Twenty‐five ROI sites were identified in the 23 patients out of which 22 correlated to the site of one of the 30 focal drivers (73%) identified in these patients. The drivers that were identified on the sequential ROI maps showed greater consistency (86.9 ± 15.1 vs 71.0 ± 19.4% of maps; *P* = 0.02), a higher recurrence rate (12.4 ± 2.7 vs 7.2 ± 0.9; *P* < 0.001) and temporal stability (3.7 ± 0.5 vs 2.3 ± 0.1; *P* < 0.001) compared with drivers that were not identified. Further to this, these drivers were more frequently associated with AF termination compared to CL slowing (termination with ablation of 17 of 22 drivers identified on sequential ROI maps vs 0 of 8 not identified on sequential ROI maps; *P* < 0.001). Table 2 demonstrates the accuracy of the ROI algorithm when using global or sequential mapping. Figure 5A‐E demonstrates a focal driver mapped to the inferolateral LA on the global wavefront maps that correlated to ROI sites identified with global and sequential mapping.

**Table 2:** Identification of focal drivers as ROI using different mapping modalities

Identifying a focal driver on the dynamic wavefront maps	Sensitivity %, 95% CI	Specificity %, 95% CI	Positive predictive value %, 95% CI	Negative predictive value %, 95% CI
ROI maps with global basket mapping	79 (68‐88)	87 (76‐94)	88 (77‐94)	79 (67‐82)
ROI maps with sequential PentaRay mapping	73 (54‐88)	99 (97‐100)	88 (70‐96)	97 (95‐99)
Only assessing drivers associated with termination	81 (58‐95)	98 (96‐99)	77 (58‐89)	98 (95‐99)

Abbreviations: CI, confidence interval; ROI, region of interest.

### 3.4 Ablation performed

The total radiofrequency (RF) time was 68.5 ± 16.4 minutes. The RF ablation time to achieve PVI was 41.6 ± 14.8 minutes and the mean RF time to target focal driver sites was 5.2 ± 3.2 minutes The average time spent at each focal driver site was 2.9 ± 1.5 minutes.

#### 3.4.1 Outcome

During an average follow‐up of 16.3 ± 3.7 months, 24 of 34 patients were free from AF/AT (70.6%) off antiarrhythmic drugs. Of the 10 with recurrent arrhythmia, six had AT and four had recurrent AF.

## 4 DISCUSSION

This is the first study to utilize a novel mapping algorithm created to identify focal drivers during endocardial mapping of AF. The CARTOFINDER system consistently identified sites of focal AT as ROI, without false positives in those patients with reentrant AT. Repetitive focal activations were identified in AF on global wavefront maps created with basket recordings, a majority of which were identified by the automated system as ROI. Application of the system to sequential PentaRay recordings showed that a majority of sites with repetitive focal activations were identified as ROI. Focal drivers that were detected on sequential PentaRay recordings were more consistent, showed greater temporal stability and a higher recurrence rate compared with drivers that were not identified as ROI. Ablation at the sites of repetitive focal activations identified as ROI on sequential PentaRay maps was more likely to terminate AF than drivers identified on global wavefront maps alone.

### 4.1 Use of the ROI algorithm to detect repetitive focal activations in AF

The ROI algorithm showed a high sensitivity and specificity in the identification of focal drivers utilizing either global basket recordings or sequential PentaRay recordings. In four patients, additional ROI sites were identified that did not correlate to focal drivers on the global maps. It is unclear whether this was a “false positive,” or a genuine focal driver that was not identified on the global wavefront map. In all four patients the ablation response at other sites was CL slowing rather than AF termination. Without having targeted the additional ROI sites prospectively, their mechanistic importance remains unclear.

Several studies describing focal drivers in persistent AF have done so through analysis of wavefront maps.^1^, ^2^, ^3^, ^4^, ^5^ However, scrutinizing multiple 30‐second dynamic wavefront maps and then reviewing these in different orientations can be time consuming. Interpreting maps has a learning curve and there may be some subjectivity when identifying drivers through visual inspection of dynamic wavefront maps. Application of the ROI algorithm to global basket recordings may facilitate interpretation of maps, speeding up the process of visual analysis by allowing the operator to focus scrutiny on a ROI and bringing some objectivity at least to the provisional identification of focal driver sites.

### 4.2 Mapping focal activations in AF

Epicardial mapping studies have demonstrated approximately two to four focal drivers per patient, albeit including single focal activations.^10^ However, in this endocardial mapping study a majority of patients had only one focal driver mapped with an average of 1.3 ± 0.4 per patient. This is consistent with the findings of previous endocardial mapping studies.^1^, ^11^ In this study a site of repetitive focal activations was defined as a driver only if there was a predefined ablation response. In contrast, in these epicardial‐mapping studies, the focal activations identified were not ablated and therefore it is unclear whether all of these were mechanistically important. Although the ablation response suggests that these drivers were not “false positives,” it is possible that further focal drivers went undetected using our methodology.

In this study focal drivers were mapped to the LA body with no drivers identified within the PVs. This is consistent with other studies where patients with an AF duration of more than 12 months (as in this study) were associated with increased extra PV drivers.^12^ It is of course possible that focal drivers at the conical PV ostia were missed by mapping the body of the LA with a spherical basket catheter. Regardless the PVs were isolated at the start and hence any drivers missed were still addressed with this strategy. It is unclear though whether a strategy targeting ROI without first isolating the PVs might leave undetected PV foci unaddressed.

### 4.3 Identification of repetitive focal activations on sequential mapping

Sequential high‐resolution epicardial mapping has demonstrated wavefronts arising from a focus meeting similar criteria to that used by the ROI algorithm.^11^ Focal activations have been identified on epicardial mapping in AF in other studies.^13^ It has been argued that other epicardial mapping studies that have failed to identify focal drivers during sequential mapping^14^ may have been hindered by insufficient LA coverage or too limited a recording period. This is the first study to systematically investigate whether focal drivers can be identified through sequential endocardial mapping in vivo. There was an increased likelihood of focal drivers being identified on sequential mapping if the driver showed greater consistency, higher temporal stability and recurrence rate. Furthermore, ablation of focal drivers identified as ROI on sequential mapping was more likely to terminate AF than those not identified in this way. This is intuitive; the more often a focal driver occurs the more likely it is to (a) be mechanistically important and (b) be detected. Focal drivers may be more stable than drivers with rotational activity.^2^, ^3^, ^4^ Furthermore, the core of rotational drivers can occupy a large area^2^ that may be difficult to map with sequential recordings. Therefore, sequential mapping of localized drivers in AF may lend itself better to identification of focal drivers than rotational activation patterns. Further attempts to identify these mechanisms using different methodologies will clarify this.

## 5 LIMITATIONS

One of the study limitations is the small patient numbers. However, having assessed 35 focal drivers across 139 CARTOFINDER maps, these data have allowed effective evaluation of the ROI algorithm with simultaneous and sequential mapping.

The study aim was to validate the ROI algorithm in the detection of focal drivers and apply this to AF. However, at the time of writing the ROI algorithm could not be applied prospectively in real‐time. Further work is needed to apply the ROI algorithm prospectively and study the ablation response guided by this and ultimately study the clinical impact in a larger cohort.

## 6 CONCLUSIONS

The ROI algorithm can effectively identify focal sources in AF using either basket or sequential PentaRay recordings. The ablation response of these focal sources suggests that they are mechanistically important and may be a viable therapeutic target in persistent AF ablation. Prospective studies targeting these sites are needed to evaluate the clinical impact.

## Supporting information

Supporting information

Supporting information

Supporting information

Supporting information
