# Supplementary material for: Automated detection of repetitive focal activations in persistent atrial fibrillation: Validation of a novel detection algorithm and application through panoramic and sequential mapping
Source: J Cardiovasc Electrophysiol. 2018 Oct 14;30(1):58–66. doi: 10.1111/jce.13752 (PMC6378609; doi:10.1111/jce.13752)
Supplement: Supplementary file 2 — Supporting information [file JCE-30-58-s002.docx]

*Supplemental Table 2- Demonstrates focal driver correlation to ROI sites on simultaneous and sequential mapping, driver characteristics and ablation response*

| Patient ID | Focal driver site | Correlation with  ROI^Ţ^ on ROI on  simultaneous sequential  mapping mapping | | Consecutive repeats in 30sec  mean ± SD | Recurrence  in 30sec  mean ± SD | Reproducibility  % of CF^Ŧ^ maps with driver | Ablation response |
| --- | --- | --- | --- | --- | --- | --- | --- |
| 1 | Postero-inferior lateral  Posterior roof | | Yes Yes  Yes Yes | 3.8±0  3.8 ± 0.6 | 13±0  12±1 | 75  75 | AT  SCL^♯^ |
| 2 | Mid roof | | Yes Yes | 3.3±1.1 | 12.7±3.5 | 80 | SCL |
| 3 | Mid anterior  Lateral | | Yes Yes  Yes No | 3.5±1.2  2.1±0.3 | 17.7±4.0  8.0±0.6 | 100  100 | AT  SCL |
| 4 | Roof  Distal CS | | Yes Yes  Yes No | 3.8±0.6  2.3±0.4 | 13.3±0.6  8.0±1.7 | 100  100 | AT  SCL |
| 5 | Roof | | No N/A | 3.3±1.1 | 6.3±0.6 | 67 | AT |
| 6 | Roof  Antero-septum | | Yes Yes  No No | 2.8±0.9  2.3±0.5 | 11.0±0  6.7±0.7 | 100  67 | AT  SCL |
| 7 | Lateral | | Yes Yes | 3.3 ±1.3 | 18.0±7.7 | 67 | SR |
| 8 | Mid roof | | Yes Yes | 2.9 ±0.9 | 15.5±0.7 | 67 | AT |
| 9 | Mid roof | | Yes N/A | 2.4 ±0.5 | 7.0 ±1.0 | 67 | AT |
| 10 | Lateral | | Yes Yes | 3.3±1.1 | 10.5±0.5 | 100 | AT |
| 11 | Posterior LAA^§^  Postero-inferior | | Yes Yes  Yes Yes | 3.2±0.7  3.4 ±0.6 | 10.3±0.5  9.3±0.5 | 100  100 | AT  SCL |
| 12 | Lateral | | No N/A | 2.4±0.5 | 10.5±0.5 | 67 | SCL |
| 13 | Anterior LAA | | Yes N/A | 2.8±0.9 | 10.5±0.5 | 100 | AT |
| 14 | Posterior roof  Antero-septum | | Yes Yes  Yes Yes | 3.5±0.7  4.2±0.7 | 10.0±1.4  14.7±0.5 | 67  100 | SCL  AT |
| 15 | Anterior LAA | | Yes Yes | 3.8±0.7 | 10.3±0.5 | 100 | SR |
| 16 | Mid anterior | | Yes N/A | 2.4±0.5 | 5.5±0.5 | 67 | SCL |
| 17 | LAA | | Yes Yes | 3.7±0.7 | 11.5±1.5 | 67 | AT |
| 18 | Roof/  LAA | | Yes Yes | 3.9±0.7 | 10.3±0.5 | 67 | AT |
| 19 | Roof | | Yes Yes | 3.1±0.7 | 8.3±0.5 | 100 | SCL |
| 20 | Roof/  LAA  Postero-lateral | | No No  Yes Yes | 2.3±0.9  4.0±0.5 | 8.5±1.2  16.5±1.1 | 50  100 | SCL  SR |
| 21 | Roof/  LAA | | Yes No | 2.2±0.4 | 6.0±0 | 50 | SCL |
| 22 | LAA/  LPV^¶^ ridge | | Yes Yes | 4.1±1.3 | 12.0±2.0 | 80 | SR |
| 23 | Low Anterior | | Yes Yes | 3.6±1.1 | 10.0±0 | 100 | SR |
| 24 | Roof | | Yes No | 2.3±0.4 | 7.0±1.0 | 67 | SCL |
| 25 | Mid Anterior | | Yes No | 2.3±0.5 | 6.0±0 | 67 | SCL |
| 26 | Lateral | | Yes Yes | 4.5±0.6 | 12.0±0 | 67 | AT |
| 27 | Roof | | Yes Yes | 4.7±0.8 | 13.7±0.5 | 100 | AT |
| 28 | Postero-lateral | | No No | 2.2±0.6 | 7.0±0 | 67 | SCL |

^Ţ^ROI- Region of interest

^Ŧ^CF- CARTOFINDER

^§^LAA- Left atrial appendage

^¶^LPV- Left pulmonary vein

^♯^SCL- Slowing of cycle length
