# Supplementary material for: Automated detection of repetitive focal activations in persistent atrial fibrillation: Validation of a novel detection algorithm and application through panoramic and sequential mapping
Source: J Cardiovasc Electrophysiol. 2018 Oct 14;30(1):58–66. doi: 10.1111/jce.13752 (PMC6378609; doi:10.1111/jce.13752)
Supplement: Supplementary file 3 — Supporting information [file JCE-30-58-s003.docx]

SUPPLEMENTAL METHOD

*CARTOFINDER mapping with basket catheters*

As described in previously published work the CARTOFINDER mapping system was used to create global dynamic wavefront maps through panoramic mapping with whole-chamber basket catheters (Constellation, Boston Scientific, Natick, MA and FIRMap Abbott, CA, USA) The system does this through creating global activation maps through using local activation times obtained from the basket catheter electrodes that were in contact, allowing for simultaneous mapping of the LA. These are achieved through annotating atrial signals during a 30-second unipolar recording obtained through referencing to Wilson’s central terminal (3). The CARTOFINDER mapping system can also be used during sequential mapping of the LA through creating dynamic wavefront maps using local activation times obtained from a PentaRay catheter. The electrograms recorded during the simultaneous and sequential mapping can then be used to create region of interest (ROI) maps. Unipolar signals were filtered at 2-240Hz, with a notch filter at 60Hz. CARTOFINDER annotates unipolar electrograms using wavelet analysis (3, 9). Electrograms and annotation can be reviewed and annotated in an open format as described previously (9). No further filtering or processing was applied. No other settings were specified. Filtering was kept consistent between all patients.

The basket catheters were sized in accordance to the LA transverse or longitudinal diameter that was obtained from the transthoracic echocardiogram performed on the day of the procedure. The catheter was positioned in the LA through an 8Fr Mullen’s (Cook Medical, In, USA) or 8.5Fr SL1 sheath (Daig Medical, MN) under fluoroscopic guidance. The basket catheter was manipulated until optimal coverage and contact was achieved. A recording was then taken with the CARTOFINDER mapping system with a minimum of 2 recordings per patient with the catheter repositioned between recordings. Following this, all patients underwent wide area circumferential ablation to achieve pulmonary vein (PV) isolation using a Thermocool© SmartTouch™ or Thermocool© SmartTouch Surround Flow™ catheter (Biosense Webster, Inc, CA) for ablation. Further CARTOFINDER mapping was then performed with the basket catheter post-PV isolation. These dynamic wavefront maps were then prospectively reviewed by the two operators that performed the case with the aim to identify and ablate localized focal drivers that met the study criteria.

*ABLATION STRATEGY*

Ablation at the driver site was performed 20 minutes post-PV isolation to minimize the impact of PV isolation on the ablation response seen at the driver site. Ablation at the driver sites was delivered with a contact force of 5-40 g, with a power of 30-40W. The driver sites were then targeted with ablation initially performed at the center of the driver with further consolidating lesions around the center. Ablation was continued until 1) a pre-defined ablation response was achieved 2) no signal was present at the driver site or 3) 5min of ablation had been performed at each site.

Beyond isolating PVs and targeting potential drivers no additional ablation was performed in AF. If the AF organised into an AT this was mapped and ablated.
