# Supplementary figures and images for: Automated detection of repetitive focal activations in persistent atrial fibrillation: Validation of a novel detection algorithm and application through panoramic and sequential mapping
Source: J Cardiovasc Electrophysiol. 2018 Oct 14;30(1):58–66. doi: 10.1111/jce.13752 (PMC6378609; doi:10.1111/jce.13752)

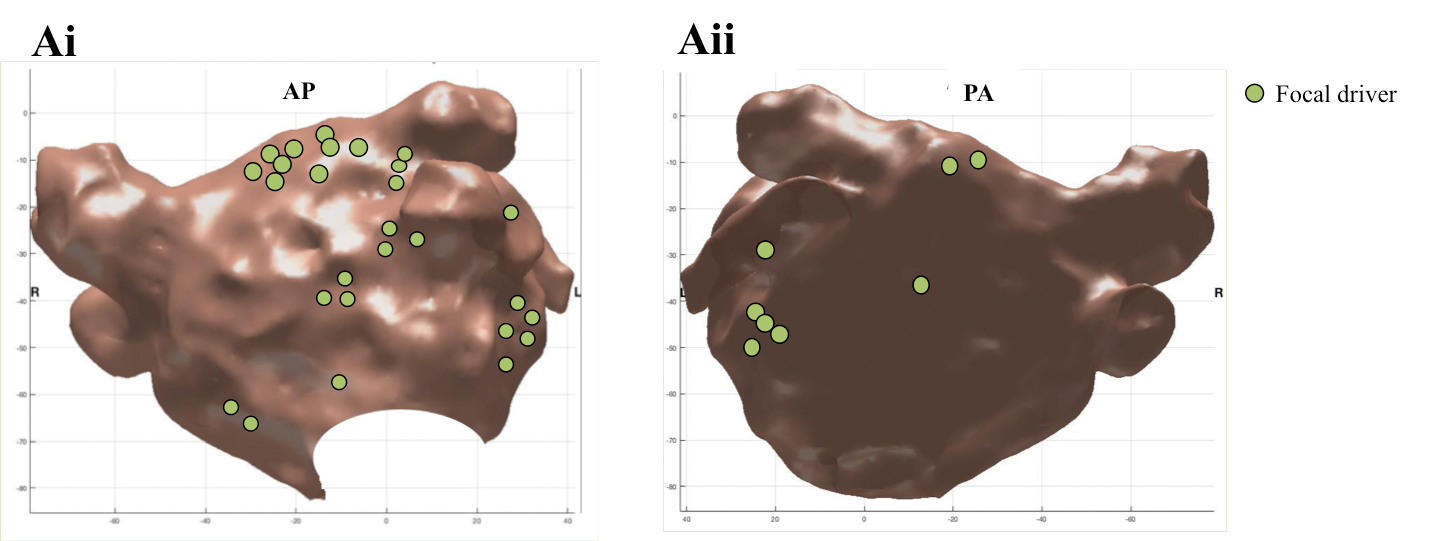

Supplement: Supplementary file 4 — Supporting information [file JCE-30-58-s004.tif]
